# Mesoporous Silica as a Drug Delivery System for Naproxen: Influence of Surface Functionalization

**DOI:** 10.3390/molecules25204722

**Published:** 2020-10-15

**Authors:** Lukáš Žid, Vladimír Zeleňák, Miroslav Almáši, Adriana Zeleňáková, Jaroslava Szücsová, Jozef Bednarčík, Monika Šuleková, Alexander Hudák, Lucia Váhovská

**Affiliations:** 1 Department of Inorganic Chemistry Faculty of Science, P.J. Šafárik University, Moyzesova 11, SK-041 54 Košice, Slovakia; lukas.zid@student.upjs.sk (L.Ž.); miroslav.almasi@upjs.sk (M.A.); 2 Institute of Physics, P. J. Šafárik University, Park Angelinum 9, 04001 Košice, Slovakia; adriana.zelenakova@upjs.sk (A.Z.); jaroslava.szucsova@student.upjs.sk (J.S.); jozef.bednarcik@upjs.sk (J.B.); 3 Department of Chemistry, Biochemistry and Biophysics, Institute of Pharmaceutical Chemistry, The University of Veterinary Medicine and Pharmacy, 04181 Košice, Slovakia; monika.sulekova@uvlf.sk (M.Š.); alexander.hudak@uvlf.sk (A.H.); lucia.vahovska@uvlf.sk (L.V.)

**Keywords:** drug delivery system, nanomaterials, mesoporous silica, naproxen, prolonged release

## Abstract

In this work we describe the relationship between surface modification of hexagonally ordered mesoporous silica SBA-15 and loading/release characteristics of nonsteroidal anti-inflammatory drug (NSAID) naproxen. Mesoporous silica (MPS) was modified with 3-aminopropyl, phenyl and cyclohexyl groups by grafting method. Naproxen was adsorbed into pores of the prepared MPS from ethanol solution using a solvent evaporation method. The release of the drug was performed in buffer medium at pH 2 and physiological solution at pH 7.4. Parent MPSs as well as naproxen loaded MPSs were characterized using physicochemical techniques such as nitrogen adsorption/desorption, thermogravimetric analysis (TG), Zeta potential analysis, Fourier transform infrared spectroscopy (FT-IR), and elemental analysis. The amount of naproxen released from the MPSs into the medium was determined by high-performance liquid chromatography (HPLC). It was shown that the adsorption and desorption characteristics of naproxen are dependent on the pH of the solution and the surface functionalization of the host.

## 1. Introduction

In the early 1990s, scientists from the Mobile company discovered a highly ordered mesoporous silica MCM-41 (Mobile Composition of Matter) [1]. Their discovery started intense research that showed the exceptional properties of mesoporous silica (MPS), such as good biocompatibility, large specific surface area, controllable pore volume and particle size. The properties of mesopores, including their size and volume as well as the surface properties of MPS, can be altered depending on additives used to fabricate silica nanoparticles and/or by a simple change of preparation conditions (temperature, time, pressure, precursor type, concentration, etc.). Various types of mesoporous carriers like MCM-41, MCM-48, SBA-15, SBA-16, and SBA-12 with unique morphology, pore size, and structure are known [2,3]. The surface of the MPS can be modified and tailored by modification with metal nanoparticles, using different approaches and preparing advanced functional materials [4,5].

One of the most promising applications of MPS nanomaterials is their use as nanocontainers for drugs in drug delivery systems (DDS) [3,6,7,8]. The unique mesoporous structure of silica facilitates the effective loading of drugs and their subsequent controlled release. Active surface enables functionalization to modify surface properties or to modify surface with a target-specific ligand that allows drug transport to the desired site of action [9,10]. The tunable mesopore structure and modifiable surface of mesoporous silica nanoparticles allow incorporation of various classes of drug molecules and controlled delivery to the target sites. It has been shown that silica is able to store and gradually release therapeutically relevant drugs like antibiotics [11] or nonsteroidal anti-inflammatory drugs (NSAIDs) [12,13]. Surface-functionalized mesoporous silica nanoparticle materials can be readily internalized by animal and plant cells without posing any cytotoxicity issue in vitro [7,14] and thus can be used for designing of a new generation of drug/gene delivery systems and biosensors for intracellular controlled release and imaging applications.

Mesoporous silica DDS are particularly useful for the drug molecules with a lack of specificity and solubility. It is well known that more than 40% of new drugs identified through combinatorial screening are poorly water-soluble [15]. These drugs are characterized by low adsorption and poor bioavailability. Such drugs may lead patients to take high doses of the drug to achieve sufficient therapeutic effects. The high or frequent dosing of the drugs and their absorption in unrelated sites leads to the suboptimal concentration of bioactive agents in target sites and contributes to the restriction in therapeutic effect. This is a leading cause of adverse drug reactions, particularly for drugs with a narrow therapeutic window [16]. For example, vancomycin, antibiotic with bactericidal activity against Gram-positive bacteria, requires a high-loading dose to have a sufficient effectiveness; it was observed that more than 4 g of it can cause severe nephrotoxicity [17]. DDS based on mesoporous silica nanoparticles can overcome such drawbacks of hydrophobic drugs [18].

The first mesoporous silica-based DDS was MCM-41 material [19]. The release study of ibuprofen loaded into mesopores of MCM-41 showed sustained release over 3 days. In addition to MCM-41, different silicas were tested for drug loading and release, and, among them, the SBA-15 has been extensively reported since its two-dimensional porous structure similar to MCM-41, but larger pore size (5–10 nm in diameter) [2,20].

There are two approaches to influence the release rate of the drugs from mesopores. The first approach is based on a regulation of pore structure, particle size, and pore diameter. For example, Qu et al. demonstrated that the release rate is dependent on the particle size, which was proportional to the length of pores [21]. Furthermore, Carriazo et al. observed that the drug content itself influenced the release rate. They found that the release rate increased with decreasing drug content, and vice versa, due to the ability of the solvent to better penetrate the pores [22]. Another way of achieving sustained drug release is the modification of pores with appropriate functional groups (-NH_2_, -Cl, -CN, -SH). The surface of mesoporous silica can be modified by co-condensation (one-pot synthesis) [23,24,25], imprint coating [26,27], and grafting (post-synthesis modification) method [24,28]. In the grafting method, functional groups are covalently bonded on the surface of silica by the reaction between alkoxysilanes and hydroxyl groups on the mesoporous silica surface [29]. Balas et al. compared loading capacity and release rates of alendronate (bisphosphonate) from parent SBA-15 and MCM-41 and those modified with amine groups. They observed that the amine-modified silica has drug loading almost 3 times larger compared to unmodified silica. The authors described this behavior as a different chemical interaction between the phosphonate group in alendronate with the silanol group on the surface of unmodified materials and materials modified with 3-aminopropyl groups. Furthermore, the release rates of modified materials are lower compared to unmodified [30]. Mesoporous silica can be modified with inorganic ligands such as 12-tungstophosphoric acid (TPA). Pathan et al. studied the release properties of poorly water-soluble erythromycin from TPA modified SBA-15. They observed a slower release of erythromycin and their observations were explained by the formation of a bond between the oxygen of TPA and erythromycin [31]. 

**Figure 1:**
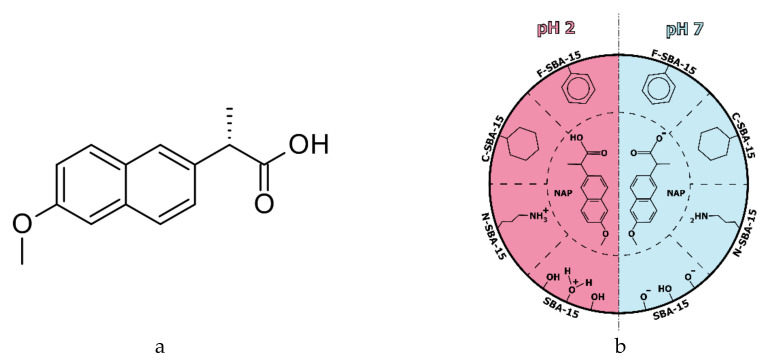
(**a**) Molecular structure of naproxen (NAP), (**b**) Schematic representation of interaction of NAP with different surface modified MPS (for the abbreviations, see the paragraph in Section 2 below).

In the present work, we investigated the influence of surface functionalization of SBA-15 to loading capacity and release rates of naproxen (see Figure 1a). Mesoporous silica (MPS) was modified with 3-aminopropyl, phenyl, and cyclohexyl groups by grafting method (see Figure 1b). Naproxen was adsorbed to the prepared MPSs from ethanol solutions using a solvent evaporation method. Naproxen (NAP) belongs to the family of nonsteroidal anti-inflammatory drugs (NSAID) of the propionic acid class. The presence of the carboxylic group improves naproxen interaction with basic amino acids like lysine in blood albumin. However, it has various side effects such as heartburn, constipation, diarrhea, ulcers, and stomach bleeding [32]. The loading of the drug into mesoporous DDS and its controlled release may not only help to the prolonged and more effective drug delivery but may also significantly reduce many undesired side-effects.

## 2. Results and Discussion

We have studied eight different SBA-15 mesoporous silica-based samples. The samples were prepared by surface modification of SBA-15 by different ligands and/or subsequent loading with naproxen molecules. In the whole paper, the following abbreviations were used: parent mesoporous silica was denoted as SBA-15. SBA-15 sample prepared by modification of SBA-15 by (3-aminopropyl)triethoxysilane (APTES), yielding the SBA-15 sample with grafted 3-aminopropyl groups, was denoted as N-SBA-15 (see Figure 1b). SBA-15 with surface modified by phenyltriethoxysilane grafting and leading to the SBA-15 sample with surface modified by phenyl group was denoted as F-SBA-15 (see Figure 1b), and SBA-15 silica modified by cyclohexyltrimethoxysilane, giving the SBA-15 sample with the surface grafted with cyclohexyl group was denoted as C-SBA-15 (see Figure 1b). Moreover, these samples were loaded by naproxen molecules (see Figure 1a). The drug-loaded samples were abbreviated as SBA-15-NAP, N-SBA-15-NAP, F-SBA-15-NAP and C-SBA-15-NAP.

### 2.1. Adsorption/Desorption of N_2_ at 77K

**Figure 2:**
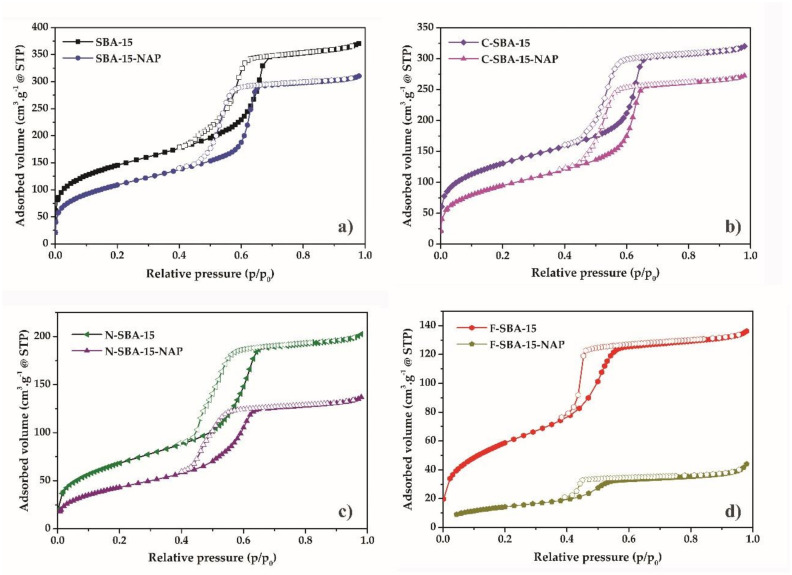
Nitrogen adsorption/desorption isotherms at 77 K for the materials (**a**) SBA-15 and SBA-15-NAP, (**b**) C-SBA-15 and C-SBA-15-NAP, (**c**) N-SBA-15 and N-SBA-15-NAP, (**d**) F-SBA-15 and F-SBA-15-NAP.

The textural properties (specific surface area, pore size, pore volume) of materials were measured by nitrogen adsorption/desorption at 77 K. All measured isotherms are depicted in Figure 2. Initially, the SBA-15 was degassed under a high vacuum, followed by a partial increase of nitrogen pressure. The micropores were filled within the relative pressures p/p_0_ = 0 − 0.1, demonstrated as a concurrent growth at the adsorption isotherm. After filling the micropores, the multilayer formation occurs from the relative pressures 0.1–0.58. Due to the capillary condensation, the mesopores filled around the relative pressure p/p_0_ = 0.65. The steady increase in the relative pressures p/p_0_ = 0.65–0.71 indicates the narrow distribution of mesopore sizes. The next increase of relative pressure did not lead to further adsorption of nitrogen, which was reflected by a plateau at the adsorption isotherm. Emptying of the mesopores delayed in comparison with their filling, and the desorption occured in the relative pressure range p/p_0_ = 0.65–0.55, leading to the formation of a hysteresis loop on the isotherms. The desorption branch of the isotherm again started to coincide with an adsorption branch below p/p_0_ = 0.5. The shape of adsorption isotherm correlated with type *IV* isotherm according to IUPAC classification, which is a typical sign of mesoporous materials with a uniform pore distribution and 1D channel structure [33]. Hysteresis loop of H1 type was observed for all samples except F-SBA-15, which isotherm had a hysteresis loop of H2(a) type. As it was shown by thermal analysis (see paragraph 2.6 below), the grafting of the phenyl ligands took place in the largest extend and sample F-SBA-15 showed the largest surface coverage. Probably, the bulkiness of the phenyl group and the large amount of the grafted ligand led to the partial blocking of the pores and creation of bottle-like shape of the adsorption/desorption isotherm [34]. All ligand functionalized and/or naproxen loaded samples showed a decrease of the amount of adsorbed nitrogen and a downshift of the capillary condensation step to lower relative pressures in comparison with parent SBA-15 sample. This reflects the filling of the mesopores of the samples by the grafted ligands and NAP molecules. 

The specific surface area determined by the BET equation for the sample SBA-15 was 528 m^2^/g and pore volume 0.496 cm^3^/g. After the modification of SBA-15 by the ligands, the BET surface area decreased to 250 m^2^/g, 216 m^2^/g, and 477 m^2^/g for the samples N-SBA-15, F-SBA-15, and C-SBA-15, respectively. Also, the pore volume decreased from the 0.496 for unmodified SBA-15 to 0.289 cm^3^/g, 0.189 cm^3^/g, and 0.435 cm^3^/g for the respective samples. The isotherms of drug-loaded samples are characteristic by the further decrease of surface area and pore volume. The textural properties of all studied samples are summarized in Table 1. 

**Figure 3:**
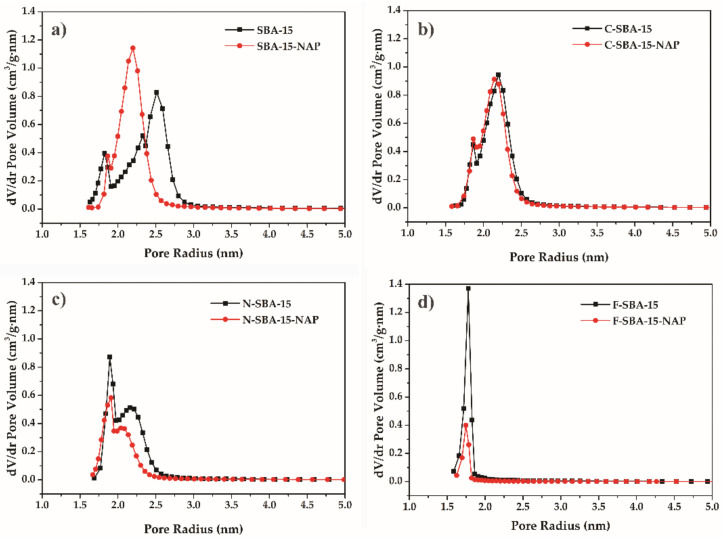
BJH pore size distribution of (**a**) SBA-15 and SBA-15-NAP, (**b**) C-SBA-15 and C-SBA-15-NAP, (**c**) N-SBA-15 and N-SBA-15-NAP, (**d**) F-SBA-15 and F-SBA-15-NAP.

The pore size of the prepared SBA-15 material calculated using the BJH method was 5.1 nm. The modification process results in a decrease of pore radius, which reflected the decrease of pore diameter by naproxen loading and modification (see Table 1). Change of the pore size distribution after modification is shown in Figure 3. 

### 2.2. HRTEM Micrographs

**Figure 4:**
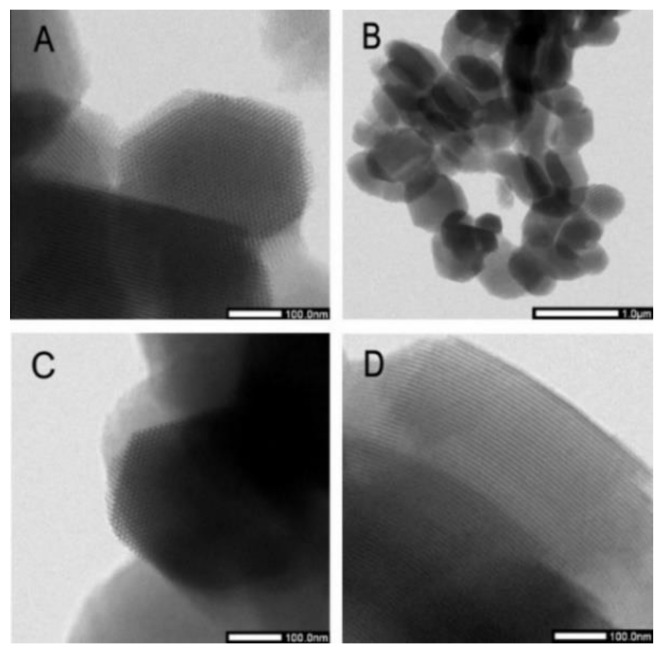
HRTEM images of SBA-15 (**A**,**B**) and C-SBA-15 (**C**,**D**).

The high-resolution transmission electron microscopy images of SBA-15 and C-SBA-15 samples at various magnifications show uniform pore size with the morphology of 2-D ordered hexagonal arrays of channels (see Figure 4). The uniform morphology of SBA-15 is well-maintained through-out the structure even after modification with cyclohexyl group. The SBA-15 and C-SBA-15 have an oval shape with the particle dimensions about 350 nm perpendicular and 650 nm parallel to the channels.

### 2.3. FT-IR Spectroscopy

**Figure 5:**
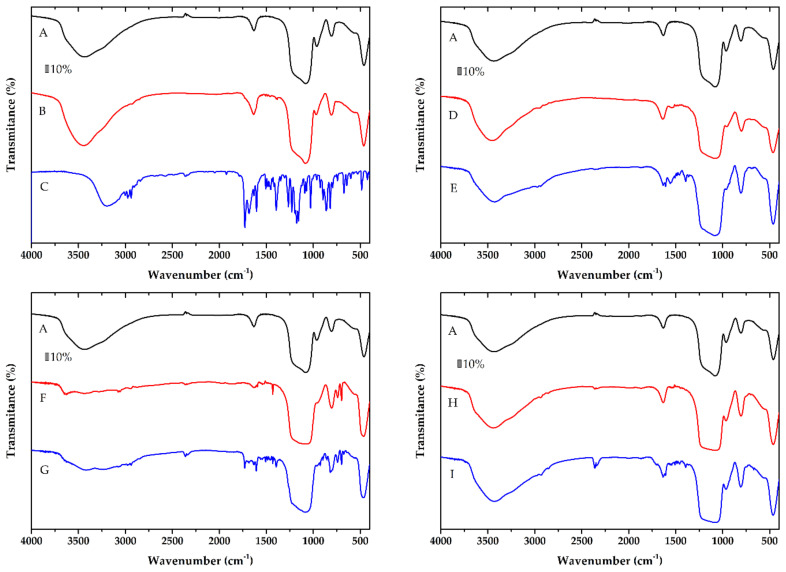
FT-IR spectra of SBA-15 (**A**), SBA-15-NAP (**B**), NAP (**C**) N-SBA-15 (**D**), N-SBA-15-NAP (**E**), F-SBA-15 (**F**), F-SBA-15-NAP (**G**), C-SBA-15 (**H**) and C-SBA-15-NAP (**I**).

The grafting of the silica by the ligands and NAP loading was evidenced by the FT-IR spectra. The FT-IR spectra of the samples before and after modification with the ligands and also before and after NAP loading were compared, and the results are shown in Figure 5. All spectra of prepared materials showed a broad band in the range of 1200–1100 cm^−1^ corresponding to an asymmetric stretch of ν_as_(Si-O-Si). The absorption band at 790 cm^−1^ corresponds to ν_s_(Si-O-Si) stretching vibration. All samples exhibit a broad band at 3400 cm^−1^ owing to the O-H valence vibration of the adsorbed water and/or surface silanol groups. All observed bands are consistent with previous studies [35]. 

The spectra of amino-functionalized sample N-SBA-15 showed small new peaks at around 1500 cm^−1^ (see Figure 5D) corresponding to the vibrations of C-H groups of the aminopropyl chain. The N-C stretch is usually observed between 1200–1000 cm^−1^, but this peak cannot be resolved owing to overlap with the asymmetric stretch vibrations of Si-O-Si band. However, the peak for the N-SBA-15 in this range is wider, suggesting a possible overlap of the bands. Furthermore, new weak peaks at 2900 cm^−1^ correspond to the C-H stretching vibrations of the aliphatic chain, demonstrating the bonding of APTES on the surface of mesoporous silica (see Figure 5D). Similarly, modification of the surface by other functional groups (Figure 5F,H) was demonstrated by the adsorption bands around 2900 cm^−1^. The band at around 1520 cm^−1^ is ascribed to the CH_2_ bending vibration. The loading of the naproxen (for its IR spectrum see Figure 5C) was reflected in IR spectra by the band of the stretching vibration ν(C=O) of the carboxylic group of naproxen at about 1720 cm^−1^, the breathing vibrations of the aromatic rings observed in the spectra in the range 1600–1500 cm^−1^. In the region below 900 cm^−1^, the γ(C–H) vibrations of naproxen were observed (see spectra in Figure 5B,E,G,I).

Thus, the grafting of organic groups and successful adsorption of naproxen in the SBA-15 may be clearly seen by comparing the spectra of the mesoporous silica, modified samples and naproxen loaded materials.

### 2.4. Small-Angle X-ray Scattering

**Figure 6:**
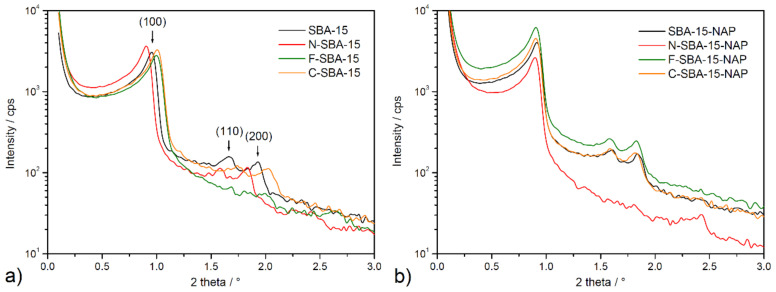
SAXS patterns of (**a**) modified and unmodified materials SBA-15, N-SBA-15, C-SBA-15, F-SBA-15, and (**b**) samples after naproxen loading SBA-15-NAP, N-SBA-15-NAP, C-SBA-15-NAP, F-SBA-15-NAP.

Prepared materials were also studied using small-angle X-ray scattering (SAXS), and obtained patterns are depicted in Figure 6. According to Figure 6, three well-resolved reflections (100), (110), and (200) can be observed on the prepared samples in the range of 2θ = 0.9–2°. The peaks can be indexed according to two-dimensional hexagonal p6mm symmetry, indicating a well-defined SBA-15 mesostructure. However, only one diffraction peak at 2θ = 1.00° is seen on functionalized F-SBA-15 material. As it was shown by thermal analysis (see paragraph 2.6 below), the grafting of the phenyl ligands took place in the largest extend and sample F-SBA-15 shows the largest surface coverage. The lower resolution of the pattern of the sample F-SBA-15 may be attributed to this factor, larger heterogeneity of the surface, and thus lower scattering power.

Based on the known values of 2θ angles, hkl indexes, and the mathematical quadratic form of the Bragg equation for the lattice with hexagonal symmetry, it was possible to calculate the unit cell parameter a (see Equation (1)) for all prepared materials that are listed in Table 2.

(1) $$a=\frac{\lambda }{sin\theta \;\sqrt{3\;}}$$

A comparison of the values for the most intensive reflection (100) for each studied sample, shows that they are small 2θ deviation in the 1.00–0.90° range for surface-modified SBA-15 samples. The 2θ values for materials after drug loading are slightly lower and vary from 0.91–0.90°. The stable position of (100) position is reflected by the similar calculated values of a cell parameter, ranging from 9.5–10.0 nm (see Table 3) for all samples. Findings from SAXS measurements confirm the well-defined porous structure of all studied materials and confirm the stability of the framework after surface modification procedure and after drug loading.

### 2.5. Zeta Potential

**Figure 7:**
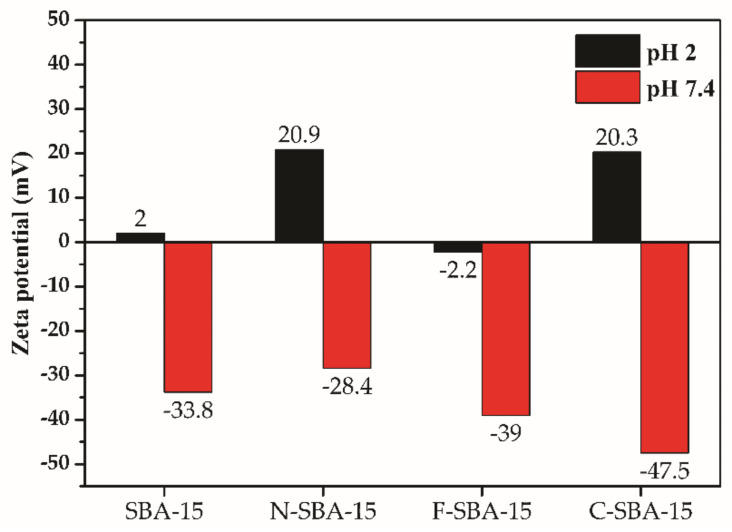
Zeta potential of SBA-15, N-SBA-15, F-SBA-15 and C-SBA-15 at pH 2 and pH 7.4.

One of the most popular uses of zeta potential data is to relate it with the colloidal stability of nanoparticles. The common guidelines classifying nanoparticles dispersions with zeta values of ±0–10 mV, ±10–20 mV, ±20–30 mV, and ±30 mV are highly unstable, relatively stable, moderately stable, and highly stable, respectively [36]. Figure 7 shows the zeta potential values for prepared samples SBA-15, N-SBA-15, F-SBA-15, and C-SBA-15 measured in two different media with pH 2 and pH 7.4. The obtained data can predict the behavior of nanomaterials under conditions immediately after administration in gastric fluid (pH = 2) with subsequent distribution through the blood into tissues (pH = 7.4). The best colloidal stability in both mediums was observed for the N-SBA-15 and C-SBA-15 nanomaterials. As can be seen, at pH 2 the sample F-SBA-15 showed different behavior in comparison with the other three samples, and negative surface charge even at low pH 2 was observed. This fact is due to large surface functionalization of the F-SBA-15 sample and diminishment of the surface -OH groups, as discussed below, in the Section 2.6.

### 2.6. Thermogravimetric Analysis

**Figure 8:**
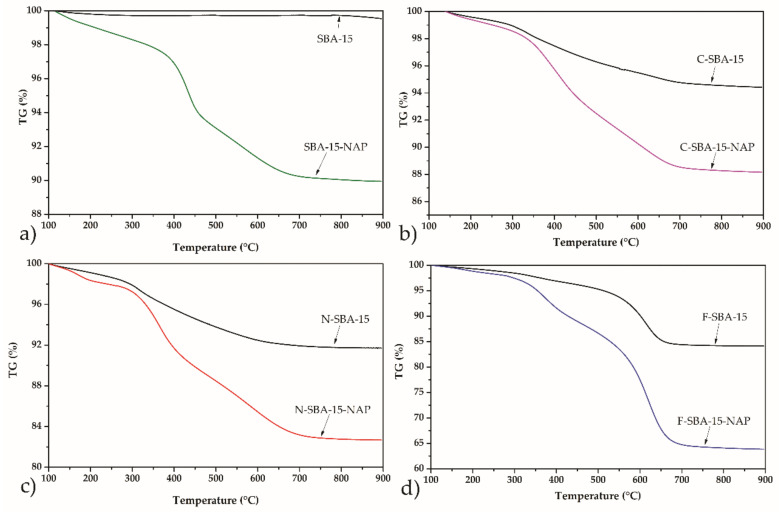
Thermogravimetric curves of (**a**) SBA-15 and SBA-15-NAP, (**b**) C-SBA-15 and C-SBA-15-NAP, (**c**) N-SBA-15 and N-SBA-15-NAP, (**d**) F-SBA-15 and F-SBA-15-NAP.

The thermal stability of the samples and determination of the amount of grafted ligands, as well as the drug loading into the modified/unmodified porous matrix, was performed by thermogravimetric analysis (TG) in air atmosphere. Figure 8 shows the TG curves of the sample SBA-15 grafted with the respective organic ligands and/or loaded with the NAP. The initial weight loss, in all samples, approximately to 250 °C, corresponds to the desorption of water or organic solvents used during the grafting (toluene) from the mesoporous matrix [35,37,38]. 

Thermogravimetric analysis of the amine-modified sample, N-SBA-15 (black curve in Figure 8c), shows a weight loss of 6.9% in the temperature range from 250 to 900 °C, corresponding to the thermal decomposition of aminopropyl ligands. For the C-SBA-15 sample, the mass loss of 4.8% was observed, and for the F-SBA-15 sample, the respective value 13.4%. From the observed mass loses, the amount of grafted ligands and surface coverage was calculated. The results are summarised in Table 3.

As can be seen, the grafting of the phenyl ligands took place in the largest extend and sample F-SBA-15 shows the largest surface coverage. This can be explained by the largest hydrophobicity of the phenyl ligand. Since the grafting was performed in hydrophobic solvent toluene, the hydrophobic ligand (phenyl) easily wetted the surface and penetrated through the pore structure, covering the largest surface area. Moreover, due to the high extend of the functionalization (surface coverage) in the F-SBA-15 sample majority of surface -OH groups were used for grafting and reacted with phenylethoxysilane. As a result, no protonation ($-OH_{2}^{+})$ occurs in low pH 2 and, in contrast to the other three samples, sample F-SBA-15 shows a negative surface charge even at this low pH (see Figure 7).

The loading of naproxen molecules into the samples was reflected by additional mass loss observed in the thermogravimetric curves (see Figure 8). From the balance of the mass loss observed for the samples grafted by the ligands and loaded with NAP in the temperature range 250–900 °C, the drug loading was calculated. The respective values are summarized in Table 3. 

### 2.7. Release Study

**Figure 9:**
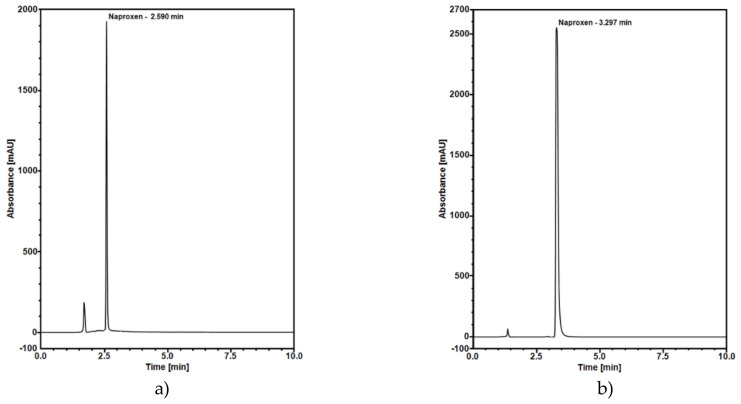
Chromatograms at optimal conditions for naproxen released at (**a**) pH 2 and (**b**) pH 7.4.

The naproxen release curves were obtained using the HPLC method. The analysis was done in the medium of pH 2 to reflect drug dissolution in the gastric fluid. Furthermore, the release test was performed at pH 7.4 that is like the pH of saliva. To optimize naproxen separation in HPLC, many compositions of mobile phases were tried. Good separation at pH 2 was obtained using a mixture of acetonitrile and water (adjusted with ortho-phosphoric acid to pH 3) at 55:45 volume ratio as a mobile phase at 25 °C (30 °C for pH 7.4) with the optimal flow set to 0.8 mL/min (1 mL/min for pH 7.4). The final chromatograms are shown in Figure 9. 

**Figure 10:**
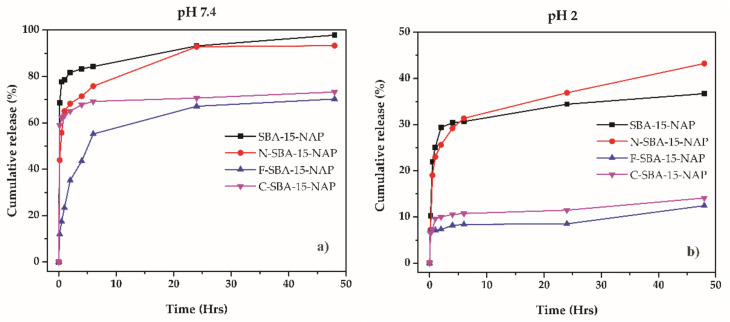
Release curves obtained using HPLC at (**a**) pH 7.4 and (**b**) pH 2 for SBA-15-NAP, N-SBA-15-NAP, F-SBA-15-NAP, and C-SBA-15-NAP.

The naproxen release profiles from prepared drug delivery systems are depicted in Figure 10. All the systems showed fast release kinetics, where the majority of the drug was released in the first 5 h. After that time, the slope of the curves changed, and between 5 and 48 h, only a negligible amount of the drug was released. Moreover, as can be seen, while at physiological pH 7.4 naproxen released from parent SBA-15 and N-SBA-15 nearly quantitively (see Figure 10a), when surface was modified by hydrophobic phenyl or cyclohexyl groups, lower amount of the drug was released. These observations can be explained as follows: 

At pH 2 (see Figure 10b), the largest amount of naproxen was released from samples SBA-15-NAP and N-SBA-15-NAP. This behavior can be explained by the surface properties and protonation of silanol and amine groups on the surface of the samples SBA-15 and N-SBA-15, which under acidic conditions, are able to form cations and $-OH_{2}^{+}$ and respectively $-NH_{3}^{+}$ [39]. On the other hand, naproxen molecules are present in the nonionized form at pH 2 (the pKa value of naproxen is equal to 4.15 and at pH 2 carboxyl group of naproxen remains nonionized). So there is no coulombic interaction between surface and drug. The nonionized form of the drug and positive surface charge of the SBA-15 or N-SBA-15 silica (see Figure 7) results in the easier release of naproxen from these two samples in comparison with C-SBA-15 and F-SBA-15 samples. In the latter samples, less than 15% of naproxen was released because of hydrophobic interactions between organic groups (phenyl- or cyclohexyl-) and naproxen. We suppose that also the formation of π-π stacking interactions between phenyl rings located on the surface of the carrier and aromatic rings of naproxen can take place. The presence of mentioned interactions was confirmed by solid-state UV measurements in our previous study [37]. However, at pH 2, the release even from the samples SBA-15-NAP and N-SBA-15-NAP was not quantitative and reached around 40%. We suppose that this is due to strong hydrogen bonding interactions between naproxen and $-NH_{3}^{+}$ and $-OH_{2}^{+}$ surface groups in the N-SBA-15 and SBA-15 silica, respectively. Moreover, in the acid media, naproxen has low solubility, so that the solution saturates before all naproxen dissolves.

At pH 7.4 the higher drug release was observed in comparison with pH 2. This can be explained by the surface charge and solubility effect. At the pH 7.4, the carboxylic group of naproxen is present in ionized form as a carboxylate anion. At pH 7.4 also silica surface is negatively charged (see Figure 7). Consequently, the interaction with the medium is preferable than the interaction with a negatively charged surface. These effects lead to the easier release of naproxen and nearly quantitative release was observed in the case of SBA-15 and N-SBA-15. However, from more hydrophobic materials (C-SBA-15 and F-SBA-15), again lower release was observed in comparison with SBA-15 or N-SBA-15 samples, similar to the acidic conditions. We suppose that it is the effect of the hydrophobicity or formation of π–π stacking interactions between phenyl rings located on the surface of the carrier and aromatic rings of naproxen and, consequently, lower solubility.

To sum up, using prepared modified mesoporous silica materials the delivery of NAP in physiological conditions is more favorable than in acidic pH. The parent or amine-modified N-SBA-15 silica showed to be more promising DDS for naproxen delivery, than phenyl o cyclohexyl modified ones. It should also be noted that surface modification can potentially influence also biological properties and cytotoxicity of prepared materials. As it was shown in our recent studies, the modification of the mesoporous silica material by grafted polar/nonpolar groups may significantly affect the compatibility with cells [37,40]. 

## 3. Materials and Methods

### 3.1. Materials

Tetraethylorthosilicate (TEOS), (3-aminopropyl)triethoxysilane (APTES) (99%), phenyltriethoxysilane (98%), cyclohexyltrimethoxysilane (99%), poly-(ethylene oxide)-poly-(propylene oxide)-poly-(ethylene oxide) triblock copolymer (Pluronic P-123) and Naproxen (>98.5%) were purchased from Sigma-Aldrich and used without further purification. Hydrochloric acid (35%), toluene (>99%), and absolute ethanol (>99.8%) were purchased from Centralchem.

### 3.2. Instrumental Methods

Infrared spectra were measured by Avatar FT-IR spectrophotometer in the range 4000–400 cm^−1^. Samples were analyzed by the KBr pellets method. Before pellets preparation, the KBr was dried in an oven at 600 °C for 3 h.

Thermogravimetric analyses (TG) were obtained at a heating rate of 10 °C/min in the air (flow rate 40 cm^3^.min^−1^)–argon (flow rate 60 cm^3^.min^−1^) atmosphere under dynamic conditions using TGA Q500 instrument.

Nitrogen isotherms of mesoporous materials were obtained at 77 K using Micromeritics ASAP 2020 plus. Brunauer–Emmett–Teller (BET) equation was used to determine the size of specific surface area, BHJ method was used to compute pore size distribution and t-plot method was used to obtain the volume of pores. 

The electro-kinetic properties of the samples were determined by dynamic light scattering (DLS) using a Malvern Zetasizer NANO-ZS (Malvern Panalytical). The particle electrophoretic mobility was measured in two different aqueous solutions with pH 2 and pH 7.4. Prior to measurement, each aqueous suspension was sonicated for 5 min. Finally, the zeta potential was calculated from the Henry equation [39].

Small-angle X-ray scattering (SAXS) experiments were done in transmission geometry using the Rigaku Ultima IV which offers parallel beam and necessary slit system (*λ* = 1.54056 Å). A small amount of powder sample was put between two kapton tapes and fixed by a metal frame. Scattered photons were collected using a NaI scintillation counter by scanning 2θ range from 0.1° up to 3° with the step size of 0.02°.

The concentration of released naproxen in solutions was determined using Dionex UltiMate 3000 RS system equipped with a diode array detector (DAD) and programmable Chromeleon Chromatography Data System, Version 7.2 (Thermo Fisher Scientific, Germany). HPLC analysis of naproxen was conducted using a ODS Hypersil C18 column (150 × 4.6 mm, 3 μm), operated isocratically. The mixture of acetonitrile and water (55:45, *v*/*v*) adjusted with ortho-phosphoric acid to pH 3 was selected as the best mobile phase. The naproxen was monitored by UV detection at 229 nm. Quantification and HPLC method validation was based on the calibration curve fitting by linear regression analysis. Linear correlation between the peak area and applied concentration was found in the concentration range of 1–60 µg/mL, as confirmed by the correlation coefficient of 0.9996. A perfect linearity was obtained in the concentration versus peak area for Naproxen drug. The mean values for the regression equation were (y = 2.8568 × −0.1348), where the y-axis was the peak area and the x-axis was the concentration of Naproxen drug in µg/mL. The sensitivity of the HPLC method was determined by LOD and LOQ. LOD and LOQ were determined by analysis of the standard of Naproxen solution. We found the LOD and LOQ were to be 0.0577 and 0.1921 µg/mL, respectively.

### 3.3. Synthesis of SBA-15 and Surface Modification

SBA-15 nanomaterial was prepared using Pluronic P-123 as a structure-directing agent. 8 g of Pluronic P-123 was added into 60 g of deionized H_2_O and 240 g of 2 M HCl. The mixture was stirred at 35 °C until complete dissolution of the surfactant followed by the addition of TEOS (16 g). The reaction medium was stirred at 35 °C for 20 h and aged at 80 °C for 24 h. The solid product was separated from a mixture by filtration, washed out with deionized water and left to dry at laboratory conditions. The surfactant was removed from mesopores by calcination at 550 °C. 

Modified SBA-15 was prepared using the grafting method. Before the experiment, the prepared SBA-15 was dried at 473 K for 2 h to remove the physisorbed water. Briefly, 1 g of dried SBA-15 was added into 50 mL of dry toluene and mixed until complete dispersion of nanoparticles was reached. The dispersion was heated under reflux, and 3 mL of the corresponding alkoxysilane was added. Subsequently, the dispersion was allowed to react for 20 h at reflux. Modified SBA-15 was cooled to room temperature, filtered off, washed with toluene, and dried at laboratory conditions. Parent SBA-15 was modified (and denoted) with 3-aminopropyl (N-SBA-15), phenyl (F-SBA-15) and cyclohexyl (C-SBA-15) groups.

### 3.4. Loading of Naproxen

Naproxen was loaded in the pores of prepared carriers by impregnation from its ethanolic solution. Each type of material was dried at 100 °C for 3 h before the experiment. 100 mg of SBA-15 was dispersed in 10 mL ethanol solution of naproxen (10 mg/mL). The mixture was stirred for 24 h using the solvent evaporation method to maximize the mass of loaded naproxen. After the adsorption step, each material was washed with 2 mL of ethanol to remove the nonadsorbed drug. This process was repeated for each material and prepared materials were denoted as SBA-15-NAP, N-SBA-15-NAP, F-SBA-15-NAP, and C-SBA-15-NAP.

### 3.5. Release of Naproxen

The release experiment was monitored in an acidic medium (KCl + HCl, pH 2) and physiological saline (pH 7.4). Prior to the experiment, the samples were analyzed to detect the amount of naproxen in the pores using the TG method. Subsequently, the samples were weighed so that each contained exactly 2 mg of naproxen in the pores. Using this method, we can minimize the effect of saturation of the solution on the release of naproxen. The mixture was stirred at 37 °C for 48 h. The concentration change in buffer medium was monitored in selected time intervals: 5 min, 15 min, 30 min, 1 h, 2 h, 4 h, 6 h, 24 h, and 48 h by HPLC.

## 4. Conclusions

It was shown that the surface functionalization of SBA-15 influences the loading and release properties of naproxen. The functionalization of the surface with more bulky and more hydrophobic ligands (cyclohexyl, phenyl) led to lower drug loading and release. Moreover, the release of the drug in two media with different pH has shown that more drug was released in neutral pH in comparison with the acidic pH. This can be explained by the acidic character of the drug itself. In the acidic environment, the surface of the silica matrices is positively charged (except the sample F-SBA-15, as discussed in the text). At acidic conditions, naproxen is neutral, in the nonionized form, which favors its passage into media. Not all drug is released, because in the acid media, naproxen has low solubility so that the solution saturates before all naproxen dissolves. In neutral media, the situation is different. Naproxen, which has pKa value equal to 4.15, has an ionized carboxylic group and penetrates to medium since its interaction with the medium is more preferable than the interaction with hydrophobic ligands and negatively charged silica surface. The material N-SBA-15 exhibits best release profiles of naproxen in both media compared to other studied materials. 

## Figures and Tables

**Table 1:** Textural properties of the samples.

Material	S_BET_ (m^2^/g)*	D_p_ (nm)*	V_p_ (cm^3^/g)*
**SBA-15**	528	5.1	0.496
**SBA-15-NAP**	398	4.9	0.433
**N-SBA-15**	250	4.7	0.289
**N-SBA-15-NAP**	159	4.5	0.194
**F-SBA-15**	216	3.7	0.189
**F-SBA-15-NAP**	53	2.4	0.065
**C-SBA-15**	477	4.9	0.435
**C-SBA-15-NAP**	346	4.8	0.390

*Abbreviations: S_BET_ = specific surface area in m^2^/g determined by BET method. D_p_ = pore diameter in nm. V_p_ = pore volume in cm^3^/g determined by BJH theory.

**Table 2:** Unit cell parameters of the studied silica materials.

Sample	2θ/°	a/nm
SBA-15	0.96	9.7
N-SBA-15	0.91	10.0
F-SBA-15	1.00	9.5
C-SBA-15	1.00	9.5
SBA-15-NAP	0.91	10.00
N-SBA-15-NAP	0.90	10.00
F-SBA-15-NAP	0.91	10.00
C-SBA-15-NAP	0.90	10.00

**Table 3:** Ligand and drug loading in the respective samples.

Material	m_grafted ligand_ (mg/g)	n_grafted ligand_ (mmol/g)	Surface Coverage (μmol.m^−2^)
**N-SBA-15**	68.5	1.159	2.194
**C-SBA-15**	47.5	0.745	1.411
**F-SBA-15**	144.8	1.854	3.511
**SBA-15-NAP**	80*	0.347 **	-
**N-SBA-15-NAP**	90*	0.391 **	-
**C-SBA-15-NAP**	62.7 *	0.272 **	-
**F-SBA-15-NAP**	197.5*	0.858 **	-

* weight of adsorbed naproxen per gram; ** mmol of adsorbed naproxen per gram.
